# The Dental Filling

**Published:** 1895-08

**Authors:** Joseph Head


ARTICLE VII.

### THE DENTAL FILLING.

BY DR. JOSEPH HEAD.

Dr. Head reviewed the different substances that had been in vogue among dentists at different times, and showed their various merits and defects. The main trouble with teeth fillings is caused by the modern bugbear, the bacillus. Gutta percha, which was once used, forms a regular hotbed for bacteria, which it turns out in a very short time in incredible quantities. Amalgam fillings are open, more or less, to the same objection. This and cohesive gold are the only materials which are supposed to exclude bacteria, although such supposition is not yet confirmed. The fact that fillings can leak bacteria and still preserve the teeth would seem to falsify the fundamental principles of dental anæsthetics, but the saving clause, as Dr. Head pointed out, is that with a good filling the number of the bacteria is greatly reduced, and the presence or absence of a suitable culture is a most important factor in the process of decay. Bacteria, like all other animal forms, may be starved to death, and this is what often happens in colonies that have found their way into the tooth. Dr. Head attributes most of the failures of dentists in tooth filling to hasty preparation of the cavity or unskillful manipulation. He believed that if the best filling available—one of gold, either of soft or cohesive foil—were perfectly adapted to good enamel edges, it would preserve the teeth as absolutely as if the original enamel remained dense and uncalcified. The filling should be polished down flush with the enamel edges and a mush of amalgam rubbed in. Gold has now been the favorite filling of dentists for fifty years, and objections to it are out of date. Dr. Head had found that the use of it on the lines he had laid down effected a great saving of trouble and disappointment both to the patient and the dentists.—*N. Y. Times.*

